# Lazarus Response to Selpercatinib for Bleeding Colic Metastasis in a Patient With RET Fusion-Positive Pulmonary Sarcomatoid Carcinoma: A Case Report

**DOI:** 10.7759/cureus.65186

**Published:** 2024-07-23

**Authors:** Federico Monaca, Emanuele Vita, Filippo Lococo, Giampaolo Tortora, Emilio Bria

**Affiliations:** 1 Medical Oncology, Fondazione Policlinico Universitario A. Gemelli IRCCS (Scientific Institute for Research, Hospitalization and Healthcare), Rome, ITA; 2 Thoracic Surgery, Fondazione Policlinico Universitario A. Gemelli IRCCS (Scientific Institute for Research, Hospitalization and Healthcare), Rome, ITA

**Keywords:** lung cancer, molecular biology, pulmonary sarcomatoid carcinoma, colic metastasis, ret fusion

## Abstract

In recent years, the widespread use of next-generation sequencing (NGS) allowed clinicians to identify and treat non-small cell lung cancer (NSCLC) efficiently with target therapy. RET inhibitors, like selpercatinib and pralsetinib, for RET rearrangements in lung cancer showed high efficacy and clinical benefit. Nevertheless, to date, the use of molecular-targeted agents has not been tested in all lung cancer subtypes. Indeed, pulmonary sarcomatoid carcinoma (PSC) remains a rare form of NSCLC, unresponsive to standard chemotherapy, and associated with extremely poor prognosis.

We report the first case of a patient affected by RET fusion-positive PSC with a bleeding colic metastasis and a consequent poor performance status who achieved a dramatic response to selpercatinib and a remarkable clinico-radiological benefit.

## Introduction

Pulmonary sarcomatoid carcinoma (PSC) is a very rare form of non-small cell lung cancer (NSCLC), which accounts for 0.1-0.4% of all lung neoplasms. PSC is characterized by epithelial-to-mesenchymal transformation, rapid tumor growth, and early metastases. Because of these characteristics and strong resistance to platinum-based chemotherapy, the prognosis is very poor with two-thirds of patients facing progression at first radiological evaluation [1]. In recent years, next-generation sequencing (NGS) enabled clinicians to identify rare oncogene alterations, like RET rearrangements, as therapeutic targets. Different tyrosine kinase inhibitors for RET fusions, such as selpercatinib and pralsetinib, have been approved and have shown durable efficacy in NSCLC. Notwithstanding the widespread use of NGS, to the best of our knowledge, no case of RET fusion has been described for a rare histotype such as sarcomatoid carcinoma. Here, we report a case of a patient with a RET fusion-positive PSC treated with selpercatinib, who experienced a very unusual colic metastasis and achieved an impressive clinical response.

This case was previously selected and presented orally for clinical case discussion at the ESMO Preceptorship on Lung Cancer 2022.

## Case presentation

**Figure 1:**
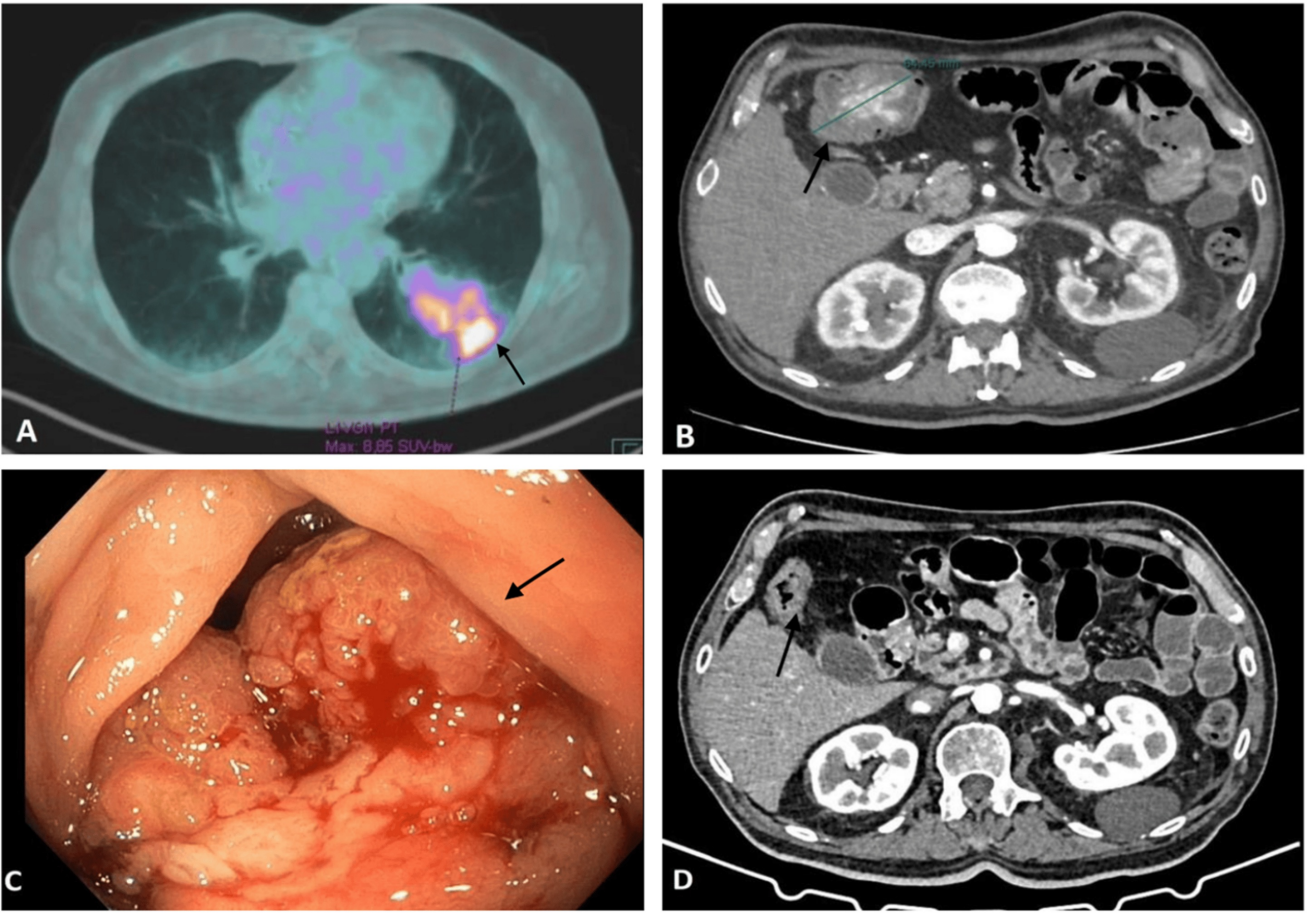
Radiological examination and endoscopic procedure. (A) PET scan showing the primary tumor before surgical resection (July 2021). (B-C) CT scan and colonoscopy showing the appearance of large bleeding colic metastasis (March 2022). (D) CT scan showing complete response of colonic metastasis after treatment with selpercatinib (September 2022).

**Figure 2:**
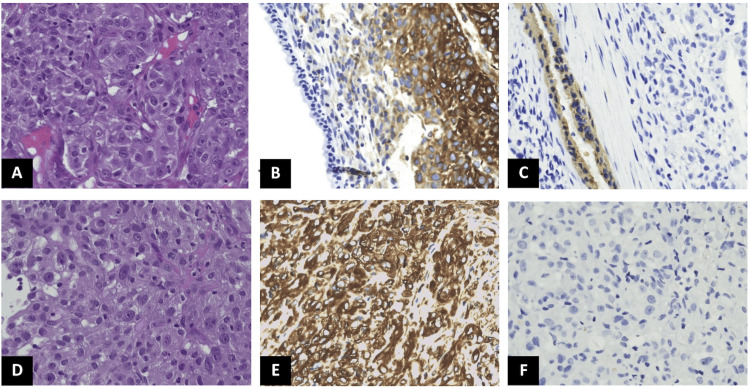
Histological examinations. Comparison of pathological examinations of primary resected tumor (A-C) and biopsy of colonic metastasis (D-F). Both samples showed malignant fusocellular cells, with plump nuclei, evident nucleoli, and abundant eosinophilic cytoplasm (A-D, hematoxylin and eosin, 400x, original magnification). Immunohistochemistry revealed diffuse strong cellular expression of vimentin (B-E) and negative expression of pankeratin (C-F) in primary tumor and colonic metastasis. Antisera to CAM5.2, epithelial membrane antigen (EMA), smooth muscle actin (SMA), desmin CD34, S100, and HMB-45 were not reactive (data not shown).

In July 2020, a 69-year-old heavy-smoker man with a history of ischemic cardiac disease (New York Heart Association class II), which resulted in decreased heart function, underwent curative surgery for a large neoplasm located in the inferior lobe of the left lung (cT4 cN0 cM0, stage IIIA; Figure 1A). Pathological and molecular characterization identified a RET-TRIM33 fusion-positive sarcomatoid tumor with low programmed death-ligand 1 (PD-L1) expression (tumor proportion score (TPS): 1-2%) (Figures 2A-2C). Adjuvant chemotherapy was excluded due to cardiological comorbidities. Unfortunately, six-month follow-up CT scans showed the appearance of multiple brain and muscular metastases. Subsequent cardiological assessment, performed with echocardiogram, confirmed a reduced ejection fraction (40%), therefore, in March 2022, a reduced upfront systemic treatment with carboplatin and gemcitabine was commenced. However, in the following weeks, the patient reported several episodes of melena leading to acute anemia (hemoglobin decreased up to 5 mg/dL) and experienced dramatic worsening of general conditions (Eastern Cooperative Oncology Group performance status (ECOG PS): 3). Abdomen CT scan revealed the appearance of a concentric lesion (64 x 42 mm) in the right colic flexure and a colonoscopy with biopsy confirmed a gross, stenotic, and bleeding neoplastic mass, mimicking a primary colorectal cancer (Figures 1B, 1C). Surprisingly, the histological examination pointed to metastasis from PSC (Figures 2D-2F). Considering the poor clinical conditions and molecular profiling, the multidisciplinary team excluded palliative surgery and decided to start salvage treatment with selpercatinib. After just one month of therapy, the patient experienced a remarkable clinical improvement with no more episodes of melena, which brought hemoglobin levels to more than 11 mg/dL and led the ECOG PS from 3 to 1. Notably, the following CT scan (September 2022) confirmed the exceptional efficacy of target therapy revealing a partial response in all sites of metastases and a complete response of the colic metastasis (Figure 1D). The patient continued to show clinical benefits and a reduction of sites of metastases till June 2023. The patient was admitted to the emergency room at the end of June for severe dyspnea. Thus, a thorax CT scan was performed and it revealed a drug-induced pneumonitis, probably related to selpercatinib. At the beginning of July, due to worsening respiratory failure, the patient died.

## Discussion

PSC is a rare, aggressive neoplasm unresponsive to chemotherapy, which has an unmet need to discover new therapeutic targets. Additionally, secondary lesions to the gastrointestinal tract from primary lung cancer are extremely rare and are usually associated with life-threatening complications [2]. To date, only one case of multiple gastrointestinal metastases from PSC has been described but data regarding its molecular profiling are lacking [3]. Comprehensive genomic profiling allowed to identify potentially targetable genomic alterations in about 30% of PSC specimens [4]. Recently, in a phase 2 trial, savolitinib showed promising activity and had an acceptable safety profile in Chinese patients with PSC harboring MET exon 14 skipping alterations, paving the opportunity for targeted treatments also in this setting [5]. However, to date, RET fusions have not been identified as a major oncogenic driver in PSC and no RET inhibitor has been tested in this histology. Indeed, the pivotal phase 1/2 trial LIBRETTO-001, which tested the efficacy of selpercatinib in NSCLC, did not include any patient with PSC [6]. Nevertheless, in this report, we demonstrate a striking radiological response leading to quick clinical benefit, just a few weeks after the beginning of treatment with selpercatinib. This evidence underlines the role of molecular-targeted agents regardless of histology, especially for neoplasms against which conventional cytotoxic agents are ineffective or are not indicated due to the general conditions of patients. Finally, to the best of our knowledge, this is the first report describing a symptomatic colonic metastasis from a RET fusion-positive PSC, followed by a marked response to selpercatinib. Further studies are required to test the role of target therapies in this specific subtype of NSCLC.

## Conclusions

We experienced a case of a RET fusion-positive PSC treated with selpercatinib. The Lazarus response to selpercatinib salvaged the patient in degraded clinical conditions and poor prognosis due to severe anemia secondary to a very unusual colonic bleeding metastasis. Our report advocates the importance of analyzing oncogene mutations to widen the use of molecular-targeted agents in this rare and poorly characterized lung cancer histology, which lacks an adequate treatment that might offer a remarkable benefit.
